# A Suspected Case of a Neonatal Monkeypox Infection With Ocular Involvement

**DOI:** 10.7759/cureus.38819

**Published:** 2023-05-10

**Authors:** Fabliha A Mukit, Emily M Louie, Hays T Cape, Shiva N Bohn

**Affiliations:** 1 Ophthalmology, University of Tennessee Health Science Center (UTHSC) Hamilton Eye Institute, Memphis, USA; 2 Ophthalmology, University of Tennessee Health Science Center (UTHSC) College of Medicine, Memphis, USA

**Keywords:** neonatal infection, orthopoxvirus, neonatal monkeypox, tecovirimat, monkeypox

## Abstract

Mpox (initially reported as monkeypox virus Clade IIb) ravaged the non-endemic world in 2022 with dermatological and systemic manifestations. The rapid propagation of this virus shed light on the scarcity of information for a virus that was first reported in 1958. We present the first probable neonatal case of mpox with ocular involvement. Ophthalmologists may be the first to diagnose mpox or be a part of the multidisciplinary team required for adequate work-up and treatment to prevent life-long sequelae in the neonatal population.

## Introduction

Previously, variations of the monkeypox virus resulted in a relatively innocuous infection that was endemic to Africa. However, the latest mpox virus has taken the world by storm in the past year due to its rapid and diverse appearance without geographic confinements. The first reported case of monkeypox was in Copenhagen in 1958, in a group of monkeys originating from Africa [1,2]. The first reported human case was of an infant from the Democratic Republic of Congo in 1970. Since its presence in the human host, cases of monkeypox have periodically appeared within African nations or originating from Africa. In 2022, reports of mpox began to arise in Europe and the United States. In July 2022, the World Health Organization declared mpox a public health emergency of international concern. While clinical outcomes have been favorable, with only 42 reported deaths in the US, vulnerable populations such as the immunocompromised, pregnant patients, and children are at increased risk for complications [3,4].

Mpox is an enveloped, double-stranded DNA virus in the genus Orthopoxviridae. This genus includes the deadly variola virus (smallpox), which caused over 300 million deaths worldwide. Additionally, it also includes the vaccinia virus, cowpox virus, camelpox virus, and ectromelia virus (mousepox). The mpox virus is divided into Clades; Clade I (formerly Congo Basin Clade) and Clade II (formerly West African Clade). Clade II is further subdivided into Clade IIa and Clade IIb. The current international outbreak is of Clade IIb [2].

As the virus was previously localized to Africa, there is a lack of discourse and research regarding the manifestations, outcomes, and effective treatment for monkeypox infection. With the international involvement of mpox virus Clade IIb, resources are now being utilized to identify the differences in the present outbreak compared to those of the past. Collective sharing of cases is now the crux to building the knowledge required to optimally treat this disease process and prevent resultant permanent sequelae. We report the first suspected neonatal mpox case with ocular involvement.

## Case presentation

**Figure 1:**
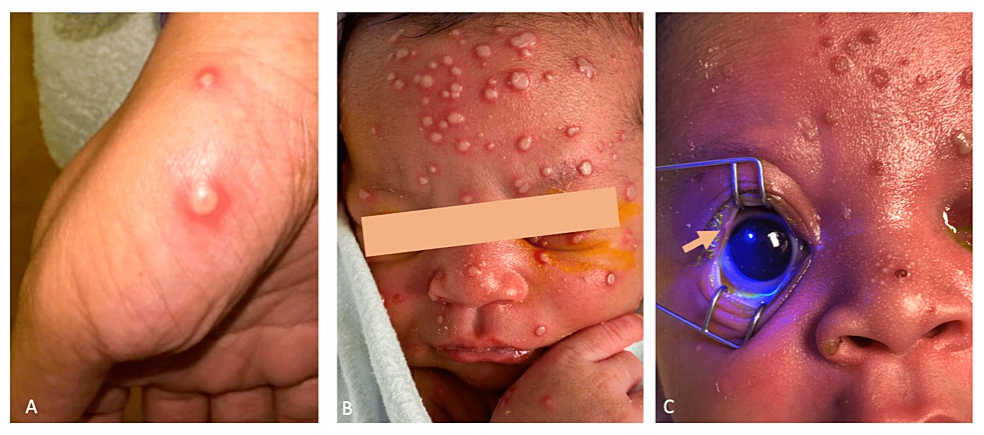
Initial presenting lesions on mother and neonate A) Mother, left thumb, on Day 2 from delivery with a pustular, umbilicated lesion; B) Frontal image from presentation in the ED on Day 10 of life with an eruption of pustular, umbilicated lesions on the forehead, glabella, cheek, and peri-oral skin; C) Day 11 of life image with lateral eyelid margin involving lesion with no fluorescein dye uptake. All lesions are at the same stage of healing. Informed consent obtained prior to release of photos.

A full-term, 10-day-old African American female, with an uncomplicated delivery and pre-natal care, presented to the emergency department (ED) due to a new-onset progressive rash and a subjective fever. The medical, ocular, family, and social history were unremarkable, except for a history of herpes simplex virus (HSV) in the patient’s mother. She was prophylactically treated with valganciclovir 10 days before delivery. Incidentally, on the day of delivery, the patient’s mother noticed a macular lesion, which progressed to a pustular lesion on her left thumb two days after delivery (Figure 1A). The mother denied any immunocompromised state or recent travel outside the city. She additionally denied any known exposure to mpox.

In the pediatric ED, the patient’s vital signs were all normal except for a fever of 100.7 F. The patient had leukocytosis of 17,100 WBCs/µL (normal range of 4,500 to 11,000 WBCs/µL) without a left shift, regular oral intake, urine, and stool output. Physical examination was significant for numerous similar-appearing circular lesions between 1 mm to 2 mm in diameter that were umbilicated, pustular with erythematous bases located on the face (predominantly on the forehead and glabella), eyelids involving the margin, abdomen, perineum, and extremities (Figure 1B). The remainder of the physical exam was normal and appropriate to the patient’s age. Due to the risk of disseminated infection, a full neonatal sepsis workup was initiated. The patient was started on empiric intravenous acyclovir, ampicillin, ceftazidime, and topical trifluridine 1% drops to both eyes.

Ophthalmology was consulted after admission due to concerns about eyelid and ocular involvement. On ophthalmic examination, the patient blinked to light in both eyes with no afferent pupillary defect or elevated intraocular pressure. A portable slit lamp examination revealed a normal anterior ocular exam. A dilated fundus exam also revealed a normal retinal exam with sharp optic nerves and no retinal lesions. The only notable exam finding was umbilicated lesions involving the bilateral eyelid and lid margin that appeared nearly identical to the lesions on the body (Figure 1C). Fluorescein staining did not reveal any corneal defect or conjunctival involvement. The trifluridine drops were discontinued, and the patient was transitioned to moxifloxacin and erythromycin drops to prevent secondary bacterial infection.

Sepsis workup returned negative for varicella zoster virus (VZV), HSV, coronavirus-19, influenza virus, and respiratory syncytial virus. A lumbar puncture with gram stain and culture of the cerebrospinal fluid was negative for any growth. The encephalitis and meningitis panels, blood, and urine cultures were also negative. Mpox polymerase chain reaction (PCR) for the patient was inconclusive, but results from the mother returned positive. Based on the disease presentation, exposure, and inconclusive mpox PCR from the neonate, the Centers for Disease Control (CDC) was consulted for management recommendations. As the mother had a positive mpox PCR with similar lesions as the neonate, the CDC recommended initiating tecovirimat (TPOXX) treatment with lubrication of the eye with bacterial coverage for suspected neonatal monkeypox.

**Figure 2:**
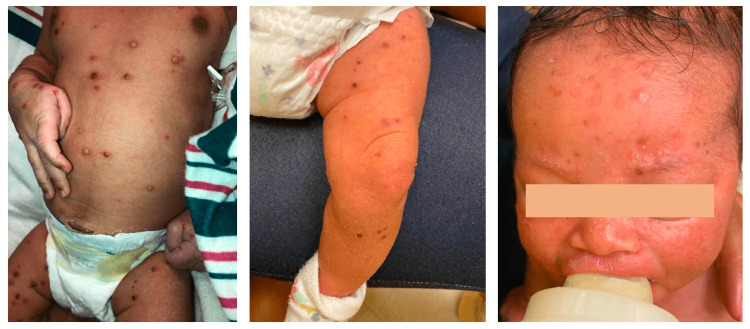
Appearance of lesions two weeks after initiating treatment with TPOXX At two weeks post-discharge, the patient had resolving crusted lesions at nearly the same stage of healing throughout the body. Informed consent obtained prior to release of images. TPOXX: tecovirimat

On day 15 of life, the patient remained afebrile with crusting and healing of the lesions. She was discharged home with TPOXX, Resinol (petroleum-based lubricant), acetaminophen, moxifloxacin eye drops, and erythromycin ointment to the eyelids. The patient followed up two weeks later in an infectious disease clinic with near-resolution of the skin lesions with no evident sequelae (Figure 2). 

## Discussion

Before the outbreak in non-endemic regions, information was scarce regarding the presentation, disease course, treatment, and outcome of the human mpox infection. According to the most recent CDC update, there have been 86,956 reported cases of mpox since January 1, 2022, of which only 1,454 cases are from endemic regions. The vast majority of non-endemic cases have been reported from the European and American (North, Central, and South) regions. The United States alone has reported 30,347 cases that resulted in 42 deaths. There is a racial predilection, with 64.07% of patients reported as Black, African American, or Hispanic persons [3].

Globally, the World Health Organization data reveals that there is a strong predisposition to mpox among men between the ages of 18 and 44 years old (78.9% of cases). The age group of 0-4 years only makes up 0.039% of cases worldwide, with a resulting lack of information on the management of this niche population. Additionally, there are increasing reports that mpox infection may be correlated with immunosuppression [5]. The neonatal immune system is a plastic, highly adaptive system that transitions from a near-sterile environment to a plethora of new organisms after delivery [6]. Due to the immaturity of the immune system, the neonate is highly susceptible to infections that can wreak havoc far more rapidly than its more mature counterparts [7].

Mpox should be considered on the differential in a neonate presenting with a vesicular rash, in addition to the traditional differential of VZV, HSV, bacterial skin infection, and bacterial sepsis. A lumbar puncture and a full sepsis workup should be performed on a neonate less than 28 days old and presenting with fever due to the high risk of mortality from disseminated infection [8]. While most mpox cases present with mild illness and fewer prodromal syndromes compared to previous outbreaks, severe complications include bronchopneumonia, gastroenteritis, encephalitis, sepsis, and ocular disease [9]. Ocular complications can be limited to periorbital and eyelid lesions, blepharitis, conjunctivitis, and keratitis; however, if not carefully monitored, corneal abrasion, corneal ulcerations, and keratitis can lead to scarring and permanent vision loss [1]. The pediatric population remains at high risk for developing amblyopia with any vision-obstructing corneal injury. Currently, only two known cases of neonatal mpox have been reported, neither of which reported ocular involvement [10,11].

The patient’s mother was the only confirmed close contact with positive mpox PCR, and she developed pustular lesions on the patient’s day two of life. Our patient’s presentation followed the expected poxvirus incubation period of 5-21 days, with the development of fever and macular lesions at day seven of life [12]. On initial evaluation, our patient had numerous pustular, umbilicated lesions associated with all pox viruses but in the same stage of healing, which is a key differentiating factor from other Poxviridae. Additionally, the ocular examination was normal for her age, except for the margin involving lesions that did not result in blepharitis, conjunctivitis, corneal abrasion, ulceration, or keratitis. Despite an indeterminate mpox PCR in our patient, mpox remains the most probable diagnosis after an extensive workup in the setting of close contact with a positive case.

Currently, no standard treatment guideline exists for mpox-related ophthalmic disease. Limited data are available regarding the efficacy of mpox treatment with antivirals, and its use has been typically reserved for those with severe disease [13]. TPOXX and the human vaccinia immune globulin intravenous (VIGIV) have been granted non-research-expanded access for the treatment of complicated mpox infections, such as those with ocular involvement. Cidofovir and its prodrug brincidofovir are Federal Drug Administration (FDA)-approved antiviral agents against cytomegalovirus that have evidence of activity against orthopoxviruses, including mpox. The CDC is currently developing an expanded-access protocol for brincidofovir use in mpox [13].

Most cases of mpox have been self-limited and managed with supportive therapy [1]. However, public health professionals have suggested that the emergence of mpox is associated with the cessation of smallpox vaccination [14]. This places neonates at a higher risk population in addition to their limited immune system. In our patient, initiating TPOXX appeared to accelerate the healing of lesions [13]. For ocular involvement, the CDC has recommended trifluridine drops, which are traditionally used for HSV keratitis [15]. However, side effects include corneal toxicity, and Trifluridine use should be limited to patients with corneal lesions and carefully monitored to balance efficacy to adverse effects. As our patient lacked corneal and conjunctival involvement, we chose to keep the eyes lubricated with antibiotic ointments to prevent the development of an abrasion and bacterial superinfection.

Mpox infection in the pediatric population is rare, and the clade IIb variant continues to mutate at a rate more than six to 12-fold greater than expected [16]. We share this suspected neonatal mpox case to continue to improve awareness and add to the developing data for management where the diagnosis is not definite. Due to the risk of severe disease, clinicians should maintain a high degree of suspicion in children and neonates presenting with a new onset vesicular rash. As guidelines regarding the treatment and prevention of mpox continue to evolve, ophthalmologists can play a critical role in recognizing and treating these cases, thereby decreasing the visual morbidity associated with this infection.

## Conclusions

Mpox in newborns is rare, and maintaining a high degree of clinical suspicion for new-onset vesicular lesions is essential for preventing long-term sequelae. In addition, monitoring for the involvement of lesions in and around the eye is crucial for preventing vision loss. To our knowledge, this is the first suspected case of neonatal mpox with ocular involvement that was determined as a diagnosis of exclusion. As the mpox outbreak progresses, we hope this case will help guide management in the future.
